# Supplementary material for: Effect of Methacrylic Acid Monomer on UV-Grafted Polyethersulfone Forward Osmosis Membrane
Source: Membranes (Basel). 2023 Feb 15;13(2):232. doi: 10.3390/membranes13020232 (PMC9967052; doi:10.3390/membranes13020232)
Supplement: Supplementary file 1 [file membranes-13-00232-s001.zip › membranes-2153773-supplementary.pdf]

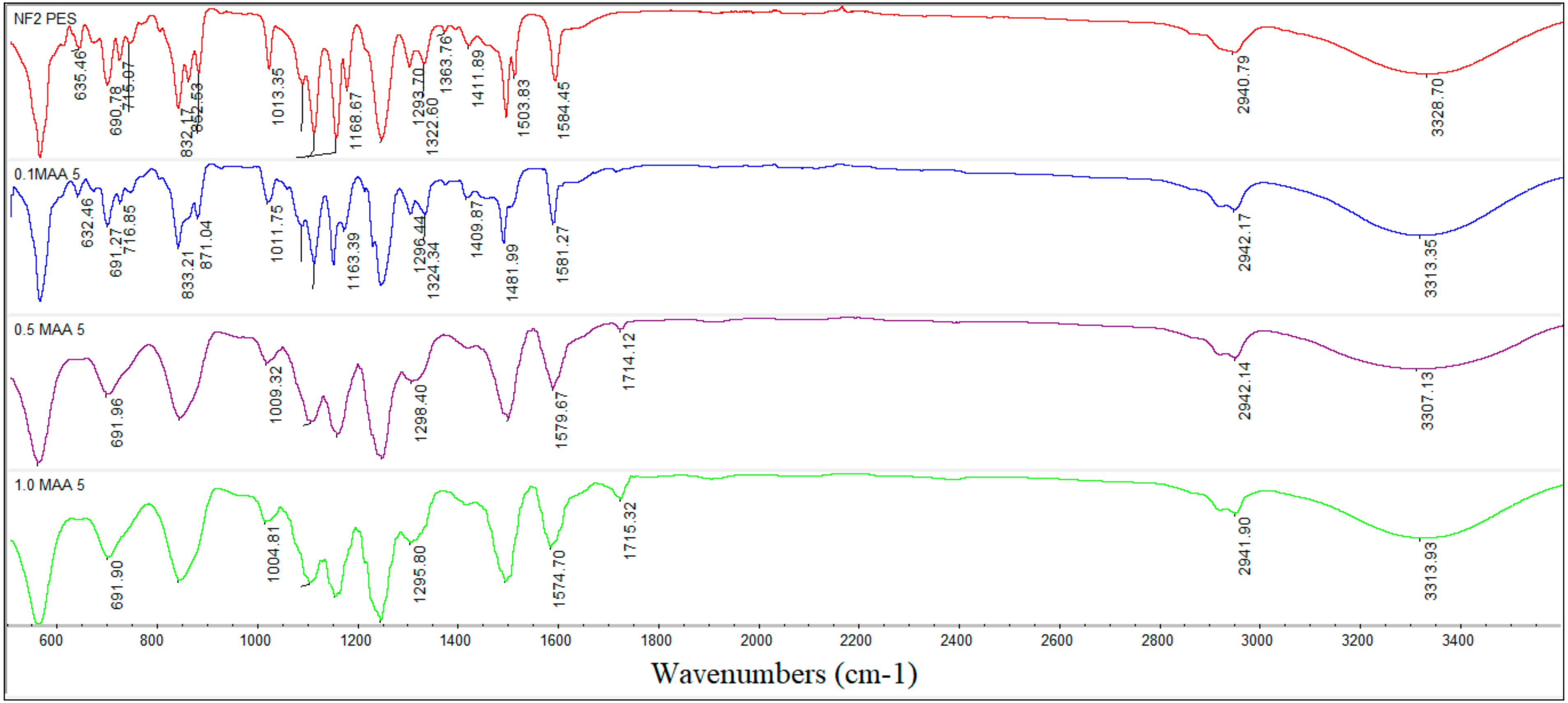

Figure S1. FTIR spectra and characteristic peaks for the unmodified NF2 PES and UV-grafted membranes 0.1MAA3, 0.5MAA3 and 1.0MAA3.

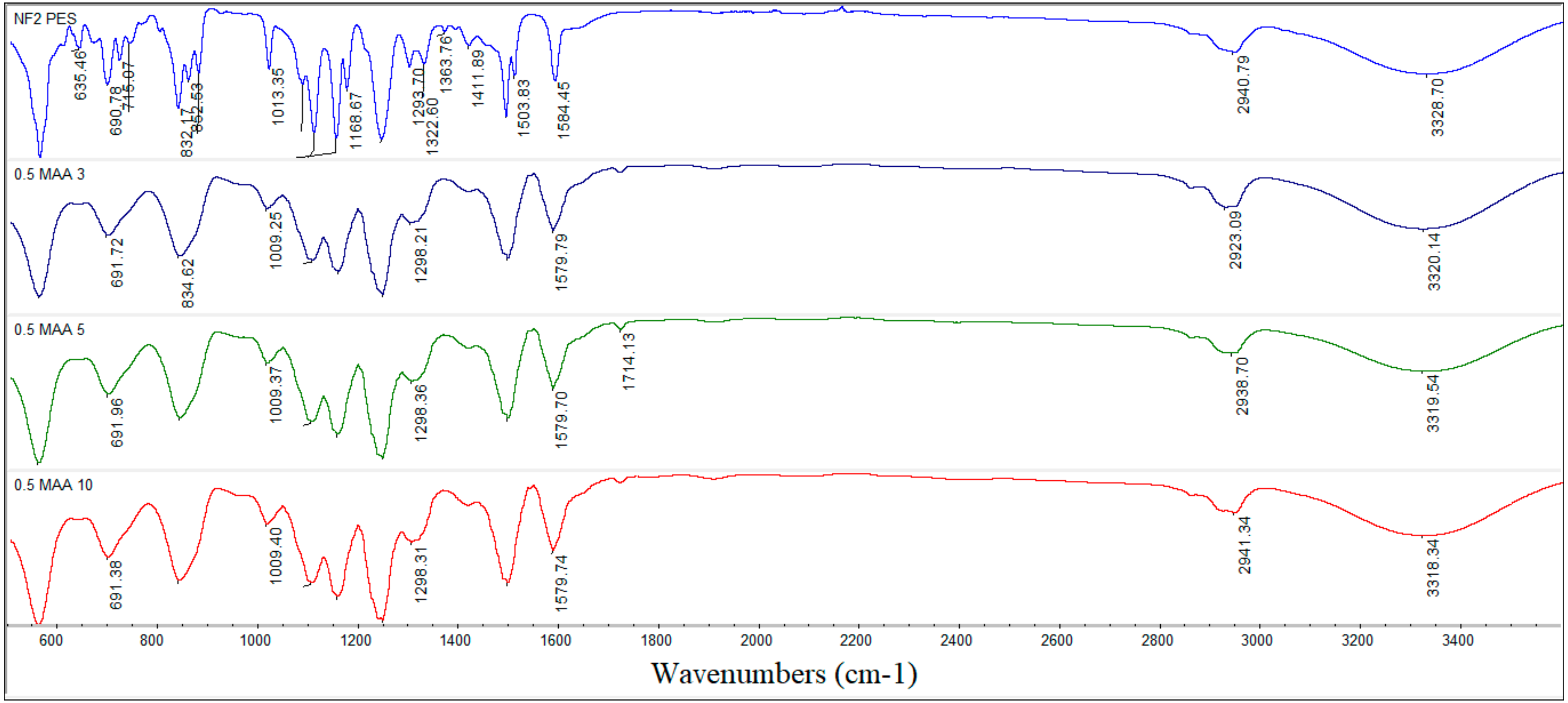

Figure S2. FTIR spectra and characteristic peaks for the unmodified NF2 PES and UV-grafted membranes 0.5MAA3, 0.5MAA5 and 0.5MAA10.
